# Treatment Switching and Drug Survival of Biologic Therapies in Psoriasis: A Real-World Italian Study Across Biologic Classes

**DOI:** 10.3390/jcm15145509

**Published:** 2026-07-14

**Authors:** Silvia Calabria, Paolo Gisondi, Marina Talamonti, Lorenzo Giovanni Mantovani, Chiara Veronesi, Luca Degli Esposti

**Affiliations:** 1 CliCon S.r.l. Società Benefit, Health, Economics & Outcomes Research, 40137 Bologna, Italy; chiara.veronesi@clicon.it (C.V.); luca.degliesposti@clicon.it (L.D.E.); 2 Section of Dermatology and Venereology, Department of Medicine, University of Verona, 37129 Verona, Italy; paolo.gisondi@univr.it; 3 Department of Dermatology, University of Rome Tor Vergata, 00133 Rome, Italy; talamonti.marina@gmail.com; 4 Research Centre on Public Health, University of Milano-Bicocca, 20900 Monza, Italy; lorenzo.mantovani@unimib.it

**Keywords:** psoriasis, biologic drugs, anti-TNFα, anti-IL23, real-world evidence

## Abstract

**Background/Objectives**: Psoriasis is a chronic inflammatory skin disease leading to substantial psycho-physical and social burden and reduced quality of life. Biologic agents have transformed its therapeutic landscape. This real-world Italian study described the pattern of treatment with biologic drugs in patients with psoriasis. **Methods**: A retrospective observational study was conducted using administrative databases from Italian Local Health Units, covering nearly 12 million individuals. The study included adults with psoriasis identified from January 2015 to March 2025 by hospitalization, co-payment exemption code, or topical anti-psoriatic prescriptions. Patients initiating a biologic drug (anti-TNFα, anti-IL12/23, anti-IL17, and anti-IL23) were selected and further analyzed in terms of treatment switching, drug survival, and healthcare resource utilization and related costs within the first year after biologic initiation, and compared. **Results**: A total of 10,270 biologic-naïve adult patients was included in the analysis (anti-TNFα *N* = 5078; anti-IL12/23 *N* = 767; anti-IL17 *N* = 2574; anti-IL23 *N* = 1851). Most patients (95.0%) starting an anti-IL23 agent did not switch. Compared with anti-TNFα, initiating an anti-IL23 inhibitor was associated with a significant reduced risk of switching (HR = 0.186; 95%CI: 0.144–0.240; *p* < 0.001). According to the cost analysis stratified by switching status, remaining on the index biologic was associated with a lower economic burden. Although differences between switchers vs. non-switchers among anti-IL23 users did not reach statistical significance (€12,052 vs. €11,406, respectively, *p* = 0.132), data support the economic advantage associated with greater treatment stability. **Conclusions**: Anti-IL23 agents showed effective, durable first-line use with potential long-term clinical and economic benefits in moderate-to-severe psoriasis.

## 1. Introduction

Psoriasis is a chronic, immune-mediated, non-transmissible inflammatory skin disease associated with a substantial physical, psychological, and social burden, leading to significant impairment in patients’ quality of life (QoL) [1].

According to the Global Burden of Disease (GBD), which reports estimates for 204 countries and territories, the number of incident psoriasis cases increased from 23.06 million in 1990 to 42.98 million in 2021 [2,3]. In Europe, prevalence rates are generally higher than in other regions, reflecting both genetic predisposition and environmental factors [4]. In Italy, psoriasis prevalence has been estimated between 1.8% and 3.1% in the general population, with regional variability reported in administrative and epidemiological studies [5].

Therapeutic management of psoriasis is guided by multiple factors, including disease severity, patient characteristics, comorbidities, and prior treatment history. Based on body surface area (BSA), Psoriasis Area and Severity Index (PASI), and overall impact on quality of life (QoL), disease severity is commonly classified as mild or moderate-to-severe [6]. While topical therapies remain the standard of care for patients with mild disease, moderate-to-severe psoriasis generally requires systemic treatment, including conventional agents (methotrexate, cyclosporine, acitretin, dimethyl fumarate), phototherapy, and biologic therapies targeting key inflammatory pathways of tumor necrosis factor alpha (TNF-α), interleukin (IL)-12/23, IL17, and IL23 [7].

Over the past two decades, the introduction of biologic agents has profoundly transformed the therapeutic landscape of psoriasis, enabling higher rates of skin clearance and substantial improvement in patient-reported outcomes. In clinical practice, treatment patterns with biologic agents encompass continuation, switching, and discontinuation, each influenced by a range of clinical and non-clinical factors [8,9]. Consequently, drug survival has emerged as a pragmatic measure to capture long-term treatment patterns and overall therapeutic performance in real-world settings [10,11,12].

Beyond short-term efficacy, optimizing therapeutic sequencing, particularly in biologic-naïve patients, has become a key issue in contemporary psoriasis management. Early selection of highly effective and durable therapies may reduce treatment instability, minimize the need for multiple switches, and potentially limit cumulative clinical and economic burden over time [13]. Recent Italian real-world evidence has identified clinical predictors associated with super-responder status to anti-IL-23p19 therapies, further supporting the importance of optimized first-line positioning strategies in biologic-naïve patients [13]. This perspective aligns with the concept of cumulative life course impairment, whereby delayed or suboptimal disease control may contribute to a progressive psychosocial and systemic burden throughout the patient’s lifespan, reinforcing the rationale for early and effective intervention in appropriate candidates [14].

Importantly, treatment patterns and switching behaviors are influenced not only by clinical factors but also by healthcare system factors, including timing of therapies market availability and reimbursement policies [15]. Anti-TNF agents were the first biologics approved for psoriasis and have historically represented the most frequently prescribed first-line biologic treatments. Over time, newer biologic classes have progressively been introduced into clinical practice, modifying treatment availability and prescribing trends [16,17]. Evaluating switching dynamics at the biologic class level may therefore provide a more robust and clinically meaningful representation of therapeutic strategies in real-world settings, reducing bias related to differences in market entry timing, access variability, and molecule-specific effects.

Despite the growing availability of real-world studies on biologic utilization in psoriasis, evidence remains limited regarding long-term switching patterns and drug survival across biologic classes within the Italian healthcare context. Therefore, this real-world analysis, based on Italian administrative healthcare databases, aimed to identify adult patients with psoriasis in Italy who initiated a biologic therapy, characterizing them in terms of demographics, comorbidities and prior treatments, and to describe real-world treatment patterns, focusing on switching between biologic therapies at class level. This study also sought to quantify healthcare resource utilization (HCRU) with the associated direct medical costs, estimated from the perspective of the Italian National Health Service (INHS), during the first 12 months after biologic initiation.

## 2. Material and Methods

### 2.1. Data Source

A retrospective observational study was conducted using data retrieved from the administrative databases of a pool of Italian Local Health Units (LHUs), collectively covering almost 12 million health-assisted individuals, with data available from January 2009 to March 2025 (at the latest, available for some LHUs). For this current analysis, LHUs databases were selected based on geographical distribution (across North/Centre/South of Italy), as well as on data completeness. Therefore, this real-world analysis utilized Italian administrative healthcare databases, ensuring access to comprehensive, high-quality linked datasets.

Specifically, the following databases were used: (i) beneficiaries database, for patient demographic data, namely, sex, age and date of death (if applicable); (ii) pharmaceuticals database, for information on reimbursed medicine, as the Anatomical Therapeutic Chemical (ATC) code, number of packages, number of units per package, unit cost per package, and prescription date; (iii) hospitalization database, including all hospitalization data, such as the main or secondary discharge diagnosis codes classified according to the International Classification of Diseases, Ninth Revision, Clinical Modification (ICD-9-CM), Diagnosis Related Group (DRG) and DRG-related charge (provided by the NHS); (iv) outpatient specialist services (OSS) database, for all information on specialist visits and diagnostic tests (date and type of provision, description of the laboratory test or specialist visit charge); (v) co-payment exemption database, for data of active exemption codes by which patients are waived from paying the contribution charge for healthcare services/treatments when specific diseases are diagnosed.

In Italy, the INHS is a universal-coverage health system that guarantees healthcare access to all inhabitants. The INHS operates at the regional level, with 20 regions responsible for implementing locally the National Health Plan. These regional authorities supervise and coordinate LHUs and the health facilities that are public or affiliated with the INHS. Each region is subdivided into LHUs, such as administrative units tasked with the delivery of healthcare services. LHUs are approximately 160, each of them responsible for healthcare planning and delivery within a defined geographic area through facilities that are public or affiliated with the INHS. The healthcare supplied is reimbursed by the LHU of residency, also in case of healthcare mobility. In larger LHUs, multiple hospitals may be managed due to the high volume of assisted individuals.

LHUs are the owners of their administrative healthcare databases, which are locally used for monitoring drug utilization, clinical outcomes, and hospital activity. These databases contain information tied to individual anonymized patient identifiers and service dates, enabling the reconstruction of detailed chronological healthcare profiles while maintaining data privacy and protection. Administrative flows capture only services provided through the INHS because they are primarily designed for reimbursement tracking between the healthcare system and providers. Indeed, the expenditure charged to the LHUs for the healthcare reimbursed by the INHS is analyzable as well in the administrative databases.

Within the administrative databases, information is recorded using a unique anonymized patient identification ID code, assigned to each subject by the Local health Units (LHUs) in full compliance with the European General Data Protection Regulation (GDPR) 2016/679, and the date of service provision. This unique ID enables consistent linkage of individual records across multiple data sources while preventing duplication. It allows electronic integration of data across the databases. This allows the collection of all available data for each individual in compliance with privacy regulations and enables the reconstruction of a complete chronological and analytical profile of healthcare services received. Therefore, the dataset used consists solely of anonymized data. All the results of the analyses were produced and presented as aggregated summaries. Approval has been obtained from the ethics committees of the participating healthcare entities.

### 2.2. Identification of Study Population

From January 2015 to March 2025 (inclusion period), adult patients (≥18 years) with psoriasis were identified through at least one of the following criteria (proxies of psoriasis diagnosis (9)): (a) at least one hospitalization with a primary or secondary diagnosis of psoriasis (ICD-9-CM code 696.1), (b) an active exemption for psoriasis (exemption code 045.696.1), or (c) at least one prescription of anti-psoriatic medications for topical use (ATC code D05A).

The index date was defined as the date of the first prescription of a biologic treatment. The characterization period included all available time before the index date, with a minimum duration of 24 months, while the follow-up period included all available time after the index date, requiring at least 12 months of follow-up for switching and drug-survival analyses. Patients were excluded if <18 years of age, had any biologic treatment for psoriasis before the index date, or had no continuous data availability in the database over the study period.

The analysis focused on patients with 24 months of data availability before the index date who started a first treatment from 2015 with TNFα, IL-12/23, IL-17 or IL-23p19 inhibitors. In detail, the following biologic drugs were considered: etanercept (ATC L04AB01), infliximab (ATC L04AB02), adalimumab (ATC L04AB04), golimumab (ATC L04AB06), and certolizumab (ATC L04AB05) among TNFα inhibitors; ustekinumab (ATC L04AC05) among IL-12/23 inhibitors; secukinumab (ATC L04AC10), ixekizumab (ATC L04AC13), and brodalumab (ATC L04AC12) among IL-17 inhibitors; and guselkumab (ATC L04AC16), tildrakizumab (ATC L04AC17), and risankizumab (ATC L04AC18) among IL-23p19 inhibitors.

### 2.3. Demographic, Clinical Characteristics and Co-Treatment at Baseline

At the index date, demographic characteristics included sex, reported as the number and percentage of males, and age (in years), summarized as mean and standard deviation (SD). Baseline clinical characteristics were assessed during the characterization period, applying a 12-month look-back before the index date for drug treatments, and using the entire available pre-index period for hospitalizations and exemption codes. Comorbidity burden was summarized through the Charlson Comorbidity Index (CCI), which assigns a score to each comorbidity, with CCI scores 1–2 indicating a mild comorbidity level, 3–4 a moderate level, and CCI scores ≥5 a severe level [18]. In addition, the presence of the following comorbidities was evaluated: PsA (ICD-9-CM: 696.0 or active exemption code: 045.696.0); IBD (ICD-9-CM: 555, 556 or active exemption codes: 009.555, 009.556); cardiovascular events (CV; ICD-9-CM: 410, 411, 413, 414, 430–438, 440, 443); hypertension (ICD-9-CM: 401, 402, 403, 404, 405 or at least 1 prescription of ATC codes: C02, C03, C07, C08, C09 or active exemption codes: A31, 031); diabetes (ICD-9-CM: 250 or at least 1 prescription of ATC code: A10); cancer (ICD-9-CM: 140–209 or active exemption code: 048); thyroiditis (ICD-9-CM: 245); dyslipidemia (ICD-9-CM: 272 or at least 2 prescriptions of ATC code: C10 or active exemption code: 025); and depression (at least 1 prescription of ATC code: N06A). Co-treatments were evaluated during the 12 months before index date and included conventional systemic drugs, namely, acitretin (ATC code: D05BB02), cyclosporin (ATC code: L04AD01), methotrexate (ATC codes: L01BA01, L04AX03), and dimethyl fumarate (ATC code: L04AX07), as well as antipsoriatics for topical use (ATC code: D05A). The number of co-treatments was stratified into classes 0–4, 5–9, and ≥10.

### 2.4. Definitions of Switching and Drug Survival

Among biologic-naïve patients, a switch was defined as the transition from the index biologic (first-line therapy) to a different biologic agent included in the analysis, corresponding to the move from first- to second line treatment. Drug survival was defined as the time from initiation of first-line biologic therapy (index date) to the first therapeutic switch. Patients who did not switch during follow-up were censored at death or at the end of data availability and classified as non-switchers. Drug survival was assessed by biologic class and using time-to-event approaches.

A subset of subjects who received at least three lines of biologic therapy were identified as multifailure patients. This subpopulation was characterized in terms of demographics, comorbidities, treatment sequences, and HCRU and related direct costs.

### 2.5. HCRU and Direct Costs

Healthcare resource utilization (HCRU) was evaluated during the first 12 months following initiation of first-line biologic therapy using administrative claims data. Resource use included the number of drug dispensations (overall and by biologic class), hospital admissions, and OSS. Analyses were restricted to patients with at least 12 months of follow-up available after biologic initiation, excluding deaths. HCRU measures were described overall and stratified by biologic class.

HCRU-associated medical direct costs were estimated from the perspective of the Italian NHS. Since administrative data were established for reimbursement purposes, they include useful information to estimate direct medical costs. Precisely, pharmaceutical costs are calculated through sum and provided as mean per capita, starting from prices of community and hospital pharmacies (inclusive of value-added tax) [19]. The in-hospital expenditure is derived by the DRG-system fee [20], which is used to calculate the reimbursed in-hospital stay costs per patient. Each DRG code corresponds to all in-hospital cares (from admission to discharge) in their entirety and complexity, without distinguishing single performed services. OSS are assessed through the current national fees, listed in the 2017 version of the “Nomenclatore tariffario” [21].

Mean annual per-patient costs were calculated for each cost component (drugs, hospitalizations, OSS) and as total integrated direct healthcare costs.

Cost analyses were conducted for the overall cohort, by biologic class, and according to switching status (switchers vs. non-switchers). To minimize the influence of extreme values, cost outliers, defined as observations exceeding the mean by more than three standard deviations, were excluded from the economic analyses.

### 2.6. Statistical Analysis

Statistical analyses were primarily aimed at characterizing patient profiles, treatment patterns, switching behavior, drug survival, HCRU and direct costs in a real-world setting. Continuous variables were reported as mean with SD and compared with two-way ANOVA; categorical variables as frequencies with percentages and compared by chi-square test.

Drug survival was evaluated using Kaplan–Meier methods and defined as the time from initiation of first-line biologic therapy to treatment switch. Patients without a switch were censored at death or end of data availability. Kaplan–Meier curves were presented with numbers at risk. Comparisons between biologic classes were performed using the log-rank test, with *p*-values reported where applicable and a two-sided significance level of 0.05.

Multivariable Cox proportional hazards models were used to estimate hazard ratios (HRs) and 95% confidence intervals (CIs) for the risk of treatment switch, primarily comparing biologic classes using a predefined reference category. Covariates included in the models were: index drug, sex, CCI, PsA, IBD, CV events, hypertension, diabetes, dyslipidemia, depression, number of conventional systemic drugs, antipsoriatics for topical use, and number of concomitant treatments.

Time-to-event analyses were restricted to patients with at least 12 months of follow-up.

HCRU and direct medical costs during the first 12 months after biologic initiation were analyzed descriptively and reported as mean values with SDs, excluding deaths and outliers.

According to Opinion 05/2014 on Anonymization Techniques drafted by the European Commission Article 29 Working Party, analyses involving fewer than 3 patients were not reported, as they could potentially allow re-identification of single individuals. Therefore, results referring directly or indirectly to ≤3 patients were reported as NI (not issuable).

Despite the substantial improvements in terms of completeness and data quality of Italian administrative data, the presence of incomplete or inconsistent data cannot be entirely excluded; therefore, if a key outcome variable was missing for a given patient, that patient was excluded from the corresponding analysis.

All analyses were performed using STATA SE, version 17.0 (StataCorp LLC, College Station, TX, USA).

## 3. Results

Within the study sample, covering approximately 20% of the Italian population, 355,161 patients with psoriasis were identified during the inclusion period. Among them, 10,270 biologic-naïve adult patients with 24 months available before the index date who initiated therapy with TNFα, IL12/23, IL17, and IL23 inhibitors during the inclusion period were included in the analysis. Specifically, 49.4% (*N* = 5078) patients initiated an anti-TNFα agent (adalimumab, *N* = 3437; certolizumab, *N* = 286; etanercept, *N* = 1195; infliximab, *N* = 160), 7.5% (*N* = 767) initiated an anti-IL12/23 agent (ustekinumab, *N* = 767), 25.1% (*N* = 2574) received an anti-IL17 agent (brodalumab, *N* = 340; ixekizumab, *N* = 872; secukinumab, *N* = 1362), and 18.0% (*N* = 1851) initiated an anti-IL23 agent (guselkumab, *N* = 692; risankizumab, *N* = 744; tildrakizumab, *N* = 415).

### 3.1. Baseline Characteristics of Adult Patients with Psoriasis by First-Line Therapy Class

As detailed in Table 1, the mean age of the overall cohort was 51.9 years at the index date, and 56.4% of patients were male. The most frequently observed predefined comorbidities were hypertension (38.9%), dyslipidemia (19.6%), and PsA (13.7%). During the characterization period, 61.3% of patients received topical anti-psoriatic treatments and 48.3% received conventional systemic therapies.

When stratified by first-line biologic class, several significant differences emerged across treatment groups. Patients initiating anti-TNFα agents showed a markedly higher comorbidity burden compared with those treated with other biologic classes, as reflected by a significantly higher mean CCI and a substantially greater proportion of patients with CCI ≥2 (*p* < 0.001). In contrast, patients treated with IL12/23, IL17, and especially IL23 inhibitors were predominantly distributed in the lowest CCI category. Regarding specific comorbidities, anti-TNFα users more frequently presented with PsA and depression (*p* < 0.001), whereas IL23-treated patients showed the lowest prevalence of PsA. IBD was more commonly observed among patients initiating IL12/23 and TNFα inhibitors (*p* < 0.001). Significant differences were also observed for cardiovascular events, diabetes, and cancer (all *p* ≤ 0.05), indicating a more clinically complex profile among patients treated with older biologic classes, particularly anti-TNFα agents. Previous treatment exposure also differed significantly between groups. Patients initiating anti-TNFα therapy had the highest proportion of prior conventional systemic drug use and a greater number of co-treatments (*p* < 0.001), suggesting a more extensive prior disease management. Conversely, patients initiating IL23 inhibitors were less frequently pretreated with conventional systemic agents.

The mean follow-up duration of the overall cohort was 3.4 years (SD 2.5). When stratified by first-line biologic class, mean follow-up was 3.6 years (SD 2.7) among patients initiating TNFα inhibitors, 5.9 years (SD 2.4) among those receiving IL-12/23 inhibitor (ustekinumab), 3.6 years (SD 2.2) among patients treated with IL-17 inhibitors, and 2.2 years (SD 1.3) among IL-23 inhibitor users.

### 3.2. Trends in Distribution of First-Line Treatments over Time

Considering the evolving therapeutic landscape of psoriasis, the distribution of first-line biologic therapies was analyzed over time according to the index year (Table 2). TNFα inhibitors represented the predominant first-line biologic class throughout the study period, accounting for 80.1% of initiations in 2015, decreasing to 36.4% in 2018, and subsequently increasing again to 68.2% in 2024. In contrast, the use of the IL-12/23 inhibitor class (ustekinumab) progressively declined over time, from 19.9–24.4% in the early years to approximately 1–2% from 2021 onwards, although the analysis does not completely capture the marketing of ustekinumab biosimilars. IL-17 inhibitors showed a marked increase following their introduction, peaking at 43.8% of first-line initiations in 2018, and then gradually declining to 14.6% in 2024. Similarly, IL-23 inhibitors were not prescribed in the earlier years of observation but demonstrated a rapid uptake following their market entry, increasing from 8.0% in 2019 to a peak of 28.0% in 2022, before slightly decreasing to 15.5% in 2024.

Overall, these trends reflect a progressive shift from early TNFα- and IL-12/23-based strategies toward greater utilization of IL-17 and IL-23 inhibitors following their introduction, followed by a recent rebalancing in prescribing patterns.

### 3.3. Analysis of Switching Trends

During the follow-up, out of 10,270 naïve patients initiating a biologic drug as first-line therapy, 7527 (73.3%) continued with the same molecule (non-switchers).

**Figure 1:**
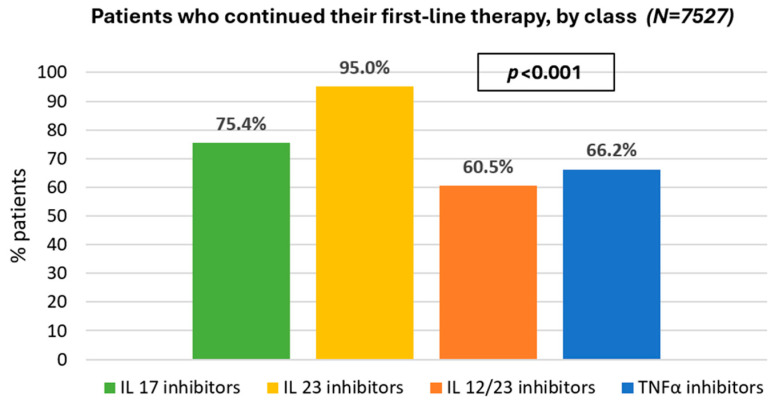
Proportion of non-switchers, by class (*N* = 7527).

As shown in Figure 1, most patients (95.0%) who started an anti-IL23 agent did not switch treatment, compared to 75.4% treated with anti-IL17, 60.5% with anti-IL12/23 and 66.2% with TNFα inhibitors.

For the remaining 2743 (26.7%) patients who switched during follow-up, a comprehensive overview of switching patterns across therapeutic classes is given in Table 3. The proportion of patients who switched therapy was lowest among those initiating IL23 inhibitor therapy (5.0%, 93 of 1851), compared with 40.2% (2044 of 5078), 39.5% (303 of 767), and 24.5% (632 of 2574) among patients initiating TNFα, IL12/23, and IL17 inhibitors, respectively (Table 3).

Switching dynamics varied substantially according to the initial biologic class. Among patients starting with TNFα inhibitors (*N* = 2044 switchers), the majority transitioned to IL17 inhibitors (58.3%), followed by IL23 inhibitors (29.2%), while a smaller proportion switched to IL12/23 inhibitor (ustekinumab) (12.5%) or another TNFα inhibitor (11.5%).

Among those initiating ustekinumab (IL12/23 inhibitor) of the 303 switchers, more than half moved to IL23 inhibitors (51.2%), whereas 26.4% switched to IL17 inhibitors and 22.4% to TNFα inhibitors, suggesting a preferential transition toward more selective IL23 targeting.

In patients treated with IL17 inhibitors in first line (*N* = 526 switchers), the predominant second-line choice was IL23 inhibitors (63.1%), followed by TNFα inhibitors (32.9%), while switching to another IL17 inhibitor or to IL12/23 inhibitor (ustekinumab) was uncommon (6.5% and 4.0%, respectively).

Among the relatively small subgroup of patients who switched treatment after initiating an IL23 inhibitor (*N* = 93), the largest majority moved to an IL17 inhibitor (70.7%). In contrast, transitions to TNF-α inhibitors represented roughly 20–30% of switches. Only a very small proportion changed to another IL23 inhibitor (1.0%) or to ustekinumab (fewer than four patients).

### 3.4. Drug Survival of First-Line Treatment by Class

**Figure 2:**
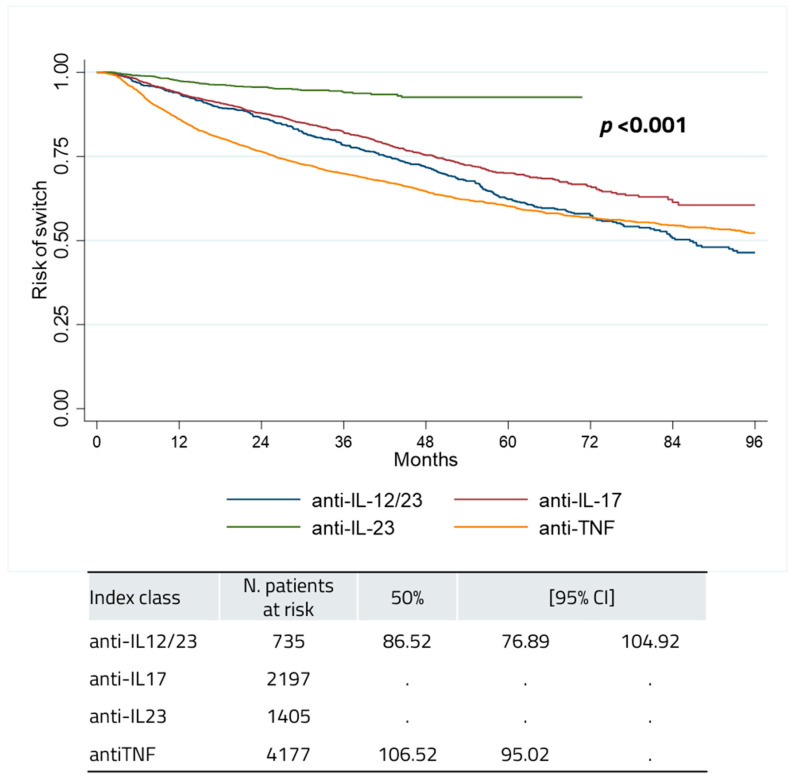
Kaplan–Meier survival analysis for drug survival (time from treatment initiation to switching to another therapy).

As shown in Figure 2, Kaplan–Meier analysis demonstrated significant differences in drug survival across biologic classes (log-rank *p* < 0.001). IL23 inhibitors showed the highest treatment persistence over time, with the lowest cumulative probability of switching throughout follow-up. Conversely, ustekinumab (IL12/23 inhibitor) was associated with the lowest drug survival, while TNFα inhibitors displayed intermediate persistence with a progressive decline, particularly within the first two years. IL17 inhibitors showed better persistence than TNFα and IL12/23 inhibitor (ustekinumab), although remaining inferior to IL23 inhibitors.

A Cox proportional hazards model (Table 4) was run using anti-TNF agents as the reference category, as they represent the first biologic class approved for psoriasis and the one with the longest market availability and historical use in clinical practice. Compared with anti-TNF inhibitors, initiation of an anti-IL23 inhibitor was associated with a markedly reduced and statistically significant risk of switching (HR = 0.186; 95% CI: 0.144–0.240; *p* < 0.001). Similarly, anti-IL17 inhibitors were associated with a significantly lower risk of treatment switching (HR = 0.641; 95% CI: 0.558–0.736; *p* < 0.001). In contrast, ustekinumab did not show a statistically significant difference in switching risk compared with anti-TNF agents (HR = 0.915; 95% CI: 0.783–1.069; *p* = 0.263).

Regarding other significant covariates, the presence of IBD was associated with a significantly reduced likelihood of switching (HR = 0.622; 95% CI: 0.453–0.854; *p* = 0.003), feasibly due to therapeutic alignment between psoriasis and IBD management. Conversely, prior exposure to conventional systemic therapies emerged as a strong predictor of switching. Compared with patients with no previous conventional systemic treatment, those exposed to one or two agents had a 20% higher hazard of switching (HR = 1.201; 95% CI: 1.102–1.309; *p* < 0.001), while patients treated with three or more conventional drugs showed more than a twofold increased risk of switching (HR = 2.305; 95% CI: 1.357–3.915; *p* = 0.002).

To assess the robustness of the results, a second multivariable Cox proportional hazards model was developed using a more recently approved biologic class as the reference category (Table 5); namely, IL12/23 inhibitor (ustekinumab). In this model, biologic class remained a strong independent predictor of treatment switching. Using IL12/23 inhibitor (ustekinumab) as the reference category, initiation of an anti-IL23 inhibitor was associated with a substantial and statistically significant reduction in switching risk (HR = 0.202; 95% CI: 0.156–0.261; *p* < 0.001), corresponding to an approximately 80% lower hazard of treatment discontinuation due to switching. Similarly, anti-IL17 inhibitors were associated with a significantly lower risk of switching compared with ustekinumab (HR = 0.698; 95% CI: 0.605–0.806; *p* < 0.001). In contrast, TNFα inhibitors did not show a statistically significant difference in switching risk compared with the reference class (HR = 1.088; 95% CI: 0.931–1.271; *p* = 0.288).

Among demographic variables, increasing age was associated with a modest but statistically significant reduction in switching risk (HR = 0.995 per year; 95% CI: 0.992–0.999; *p* = 0.011), while male sex was independently associated with improved treatment persistence (HR = 0.893; 95% CI: 0.819–0.973; *p* = 0.010).

Regarding comorbidities, the presence of IBD was associated with a significantly reduced risk of switching (HR = 0.622; 95% CI: 0.453–0.854; *p* = 0.003), whereas depression was associated with an increased switching risk (HR = 1.180; 95% CI: 1.034–1.346; *p* = 0.014). Other comorbidities were not independently associated with switching.

Finally, prior treatment exposure was a relevant predictor, as both previous use of conventional systemic therapies (HR = 1.207; 95% CI: 1.107–1.315; *p* < 0.001) and topical antipsoriatic treatments (HR = 1.236; 95% CI: 1.133–1.350; *p* < 0.001) were associated with a significantly higher hazard of switching.

### 3.5. HCRU and Associated Direct Healthcare Costs

HCRU and direct healthcare costs were analyzed during the first year of follow-up among patients with at least 12 months of observation, excluding deaths and (for costs) outliers (*N* = 8410). Overall, patients received a mean of 16.9 (SD 10.8) drug dispensations, 0.1 (SD 0.4) hospitalizations, and 6.0 (SD 5.9) OSS.

When stratified by biologic class, differences in HCRU were statistically significant but modest in magnitude. The mean number of drug dispensations was 18.7 (SD 11.0) for TNFα inhibitors, 16.9 (SD 10.5) for IL17 inhibitors, 14.2 (SD 9.8) for IL12/23 inhibitor (ustekinumab), and 13.2 (SD 9.9) for IL23 inhibitors (overall *p* < 0.001). Hospitalization rates were uniformly low (on average 0.1 in all groups; *p* = 0.009), and OSS utilization ranged from 5.2 to 6.7 visits per patient (*p* < 0.001), without clinically meaningful differences.

In contrast, direct costs showed marked and clinically relevant variation across biologic classes. Total annual direct healthcare costs were €6476.9 for TNFα inhibitors, €12,439.4 for IL17 inhibitors, €11,439.1 for IL23 inhibitors, and €13,412.5 for IL12/23 inhibitor (ustekinumab) (overall *p* < 0.001). IL17 inhibitors were associated with significantly higher total costs compared with all other classes (*p* < 0.001), while IL23 and IL12/23 inhibitor (ustekinumab) also generated significantly higher total costs than TNFα inhibitors (*p* < 0.001).

When examining cost components, differences were primarily driven by pharmaceutical expenditure. Mean annual drug costs were €5754.9 for TNFα inhibitors, €11,862.4 for IL17 inhibitors, €10,886.4 for IL23 inhibitors, and €12,916.7 for IL12/23 inhibitor (ustekinumab) (overall *p* < 0.001). Pairwise comparisons confirmed significantly higher drug-related costs for IL17, IL23, and IL12/23 inhibitors compared with TNFα inhibitors (all *p* < 0.001), likely reflecting biosimilar availability within the TNFα class.

By contrast, hospitalization costs (€394.5 TNFα; €283.5 IL17; €276.6 IL23; €223.1 IL12/23; *p* = 0.002) and OSS costs (€327.5; €293.5; €276.1; €272.7, respectively; *p* = 0.010) contributed minimally to overall cost differences.

As shown in Table 6, switching within the first year was consistently associated with higher pharmaceutical expenditure across most biologic classes, whereas hospitalization and OSS costs did not substantially differ between groups. In the cost analysis stratified by switching status, remaining on the index biologic was associated with a lower economic burden: among TNFα inhibitors, non-switchers incurred a mean total annual cost of €5446 compared with €8519 in switchers (*p* < 0.001), and a similar pattern was observed for IL17 inhibitors (€9109 vs. €10,284; *p* < 0.001), both mainly driven by increased drug-related costs. For ustekinumab (IL12/23 inhibitor), no significant differences in total costs emerged between switchers and non-switchers, suggesting a more homogeneous cost profile. Although differences did not reach statistical significance for IL23 inhibitors (€11,406 vs. €12,052, *p* = 0.132), the overall direct medical costs observed in this class, together with the markedly low switching rate, further support the economic advantage associated with greater treatment stability. Overall, these findings indicate that the direct healthcare economic burden associated with switching is predominantly driven by drug consumption.

### 3.6. Analysis of Multifailure Patients

A total of 750 patients were classified as multifailure, defined as having received at least three lines of biologic therapy (Table 7). Treatment pathways were heterogeneous but predominantly characterized by repeated use of TNFα and IL17 inhibitors, with frequent subsequent transition to IL23 inhibitors. Among patients initiating therapy with a TNFα inhibitor (*N* = 481), the most common second-line option was an IL17 inhibitor (40.1%), followed by another TNFα inhibitor (37.8%) and an IL23 inhibitor (13.7%). In this subgroup, escalation to IL23 inhibitors was observed in 20.2% of third-line sequences, while 25.2% received IL17 inhibitors again after cycling through TNFα agents.

Similarly, among patients starting with IL17 inhibitors (*N* = 177), 36.2% continued with another IL17 inhibitor in second line, and 22.0% escalated to an IL23 inhibitor in subsequent lines. In those initiating ustekinumab (IL12/23 inhibitor, *N* = 81), switching most frequently involved IL17 inhibitors (44.4%) or TNFα inhibitors (38.3%). Overall, these patterns confirm that multifailure trajectories largely involve cycling within TNFα and IL17 classes before switching to IL23 inhibitors, which frequently represent a later-line therapeutic option in more complex treatment pathways.

Among 750 patients of the multifailure cohort, there was a slight predominance of female sex (54.0%) and the mean (SD) age was 50.7 (13.1) years. Overall comorbidity burden was generally limited in this subgroup. Specifically, 57.6% of multifailure patients were classified as having a low comorbidity level according to the CCI, corresponding to a CCI score of 1.

HCRU and direct healthcare costs in the subset of multifailure patients (≥3 biologic lines) were assessed for those with at least 12 months of follow-up available, excluding deaths and (for cost analysis) outliers (Table 8). During follow-up, patients received a mean of 17.9 (SD 9.9) drug dispensations, compared with 0.2 (SD 0.3) hospitalizations and 7.1 (SD 5.9) outpatient specialist services, with no significant differences in HCRU across biologic classes. Across all classes, drugs accounted for approximately 85–90% of total direct costs, confirming that pharmacological management remains the dominant economic driver in multifailure patients.

Notably, the majority of multifailure patients originated from the TNFα class, reflecting its historical positioning as first-line therapy and widespread biosimilar use. Although TNFα initiators showed the lowest mean annual cost, the overall cost differences between biologic classes in this heavily pre-treated population were relatively limited. This pattern suggests that initial savings associated with biosimilar initiation may progressively accumulate across subsequent treatment lines as patients switch therapies, leading to a redistribution of pharmaceutical expenditures over time.

## 4. Discussion

In this large real-world analysis conducted within a sample corresponding to nearly 20% of the Italian population, biologic treatment patterns, switching dynamics, drug survival, and economic burden were comprehensively described among adult patients with psoriasis initiating biologic therapy. The findings provide robust evidence on the comparative performance of biologic classes in routine clinical practice, highlighting substantial differences in treatment persistence, switching dynamics, and associated healthcare costs. The large sample size and the long follow-up period contribute to the robustness and generalizability of the present findings. Moreover, the algorithm identifying patients with psoriasis through administrative data was previously validated and the estimates of psoriasis prevalence and biologic utilization rates were validated by the Italian experts who participated in the study (9). In addition, the analysis conducted at the biologic class level allows a more clinically meaningful interpretation of switching dynamics, reducing potential bias related to differences in market entry timing and individual drug availability.

A key result of the study was the clear gradient in treatment persistence across biologic classes. IL23 inhibitors consistently exhibited the lowest risk of treatment switching, both in Kaplan–Meier survival analyses and in multivariable Cox models, showing an approximately 80% reduction in switching risk compared with older biologic classes. This finding is consistent with multiple real-world survival analyses demonstrating that the IL23 inhibitor class exhibits superior drug survival compared with IL17 and TNF inhibitors [12,22,23]. Therefore, the lowest risk of treatment switching observed with IL23 inhibitors in our cohort is consistent with their higher drug survival rates reported in the literature. Similar results have also been reported in international registry-based studies [24]. Importantly, the greater treatment persistence observed with IL23 inhibitors may suggest improved long-term disease control, supporting their potential role in the sustained management of moderate-to-severe psoriasis over time. These findings are consistent with previous real-world evidence, including the study by Di Giulio et al., which identified predictors of super-responder status to anti-IL23 therapies among Italian patients with psoriasis [13]. In this context, our results further suggest that earlier positioning of IL23 inhibitors within the therapeutic sequence may enhance the likelihood of achieving durable and sustained responses.

The favorable persistence observed for IL23 inhibitors is biologically plausible, given their selective targeting of the IL23/Th17 axis, which plays a central role in psoriasis pathogenesis. Moreover, their convenient dosing schedules and favorable safety profiles may contribute to sustained adherence over time. Comparable persistence advantages have been previously observed in Italian real-world studies, where IL23 inhibitors were associated with lower discontinuation rates than IL17 inhibitors [25].

The therapeutic landscape of psoriasis has undergone continuous evolution over the last decade, influenced by the progressive reimbursement of biosimilars, the introduction of new biologic classes, and the expansion of dosing options within existing therapies. In particular, tildrakizumab 100 mg was reimbursed in Italy in February 2020 [26], followed by the availability of the 200 mg formulation in May 2023 [27]. More recently, the introduction of bimekizumab has further expanded treatment options. These sequential market entries and reimbursement updates have progressively reshaped prescribing patterns and switching dynamics. Consequently, real-world analyses covering extended observation periods inevitably reflect different phases of therapeutic availability and healthcare policy, meaning that the present findings should be interpreted as representative of a dynamic and evolving scenario rather than a definitive picture of the current treatment landscape [28].

Within this evolving context, tildrakizumab represents a relevant component of the IL23 inhibitor class. Real-world evidence has documented sustained effectiveness, favorable safety, and consistent drug survival. The availability of the 200 mg dose provides greater therapeutic flexibility, particularly for patients who require dose optimization to achieve adequate disease control. The available literature increasingly supports the concept of personalized biologic therapy in psoriasis, including dose-adjustment strategies aimed at maximizing long-term disease control and improving treatment persistence [29,30]. In this perspective, the introduction of the 200 mg formulation may further strengthen the long-term performance of the IL23 class observed in this study, potentially consolidating its role in achieving durable disease management.

Notably, among patients initiating treatment with TNFα biosimilars, failure to achieve adequate response and subsequent switching was associated with a 1.6-fold increase in annual pharmaceutical costs, suggesting that initial cost savings may be partially offset by the need to escalate therapy. In this perspective, treatment-related expenditures appear to be progressively redistributed across subsequent therapeutic lines in patients experiencing switching. Furthermore, the economic gap observed between switchers and non-switchers over the 12-month evaluation period is likely to widen when considering longer time horizons, as repeated treatment modifications and cumulative drug acquisition costs may amplify the overall financial burden over time. IL17 inhibitors also showed a significantly reduced risk of switching compared with older biologics, although to a lesser extent than IL23 inhibitors. This finding is consistent with previous observational analyses reporting intermediate drug survival for IL17 inhibitors, potentially influenced by faster onset of action but higher discontinuation rates due to adverse events or loss of efficacy over time [31].

TNFα inhibitors remained the most frequently prescribed first-line biologics throughout the study period, reflecting their earlier market entry and long-standing clinical familiarity. However, they were associated with higher switching rates and lower persistence, consistent with findings from other European cohorts [32,33,34,35].

Beyond treatment class, several patient-level factors influenced switching risk, as well as the choice of biologic [36]. As per the latter, biologic agents for psoriasis were found safe in patients with cardio-cerebrovascular disease [37], while screening for HBV and HCV infection is required before initiating any immunosuppressive treatment [38]. Also, the choice of the best pharmacological approach in patients with metabolic comorbidities should encompass tailored multidisciplinary interventions [39].

More specifically on switching risk, older age and male sex were associated with greater treatment persistence, a pattern previously reported and possibly related to differences in treatment expectations, adherence, or disease phenotype [40]. Notably, the observed association between prior exposure to conventional systemic therapies and increased switching risk is particularly relevant, as it suggests that treatment failure in earlier lines may significantly impact subsequent biologic stability, with patients exposed to multiple conventional agents showing more than a twofold higher risk of switching.

Interestingly, the presence of IBD was associated with a reduced risk of switching, likely reflecting therapeutic alignment between psoriasis and IBD management, where treatment choice is often more constrained and targeted to address both conditions simultaneously, thereby reducing the likelihood of switching [41]. Conversely, depression and prior exposure to conventional systemic therapies were associated with increased switching risk, highlighting the impact of psychological comorbidity and treatment-refractory disease on long-term outcomes. Indeed, this relationship is likely complex and potentially bidirectional. On one hand, underlying depressive symptoms may negatively influence treatment adherence, patient perception of effectiveness, and therapeutic satisfaction. On the other hand, repeated treatment failures and disease instability may themselves contribute to psychological distress, potentially exacerbating or triggering depressive symptoms over time. This interplay underscores the need for a multidisciplinary management approach that integrates dermatological and psychological care in patients with moderate-to-severe psoriasis [42].

Moreover, the absence of switching translated into meaningful per-patient cost savings already within the first year of treatment, primarily driven by lower pharmaceutical expenditures. This finding is consistent with previous real-world studies showing that biologic switching is a major determinant of increased healthcare costs in psoriasis, largely attributable to drug acquisition costs and treatment re-initiation rather than to hospitalizations or acute care utilization [43]. Similar cost patterns have been reported in European observational analyses, where patients who switched biologics incurred higher annual healthcare expenditures compared with persistent patients, mainly due to intensified pharmacological management [32,33]. The economic benefit associated with treatment persistence may become even more pronounced when projected over a longer time horizon (3–5 years). In this context, the superior drug survival observed for IL23 inhibitors in both registry-based and real-world studies [10,31,35] supports the hypothesis that sustained persistence may translate into cumulative cost savings for the Italian NHS, by reducing repeated therapy modifications, minimizing drug wastage, and limiting the incremental pharmaceutical expenditures associated with multiple treatment lines.

The analysis of multifailure patients revealed a small but clinically relevant subgroup characterized by repeated switches across biologic classes, predominantly involving TNFα and IL17 inhibitors before escalation to IL23 inhibitors. In multifailure patients, healthcare costs were predominantly driven by pharmacological treatments, while hospitalizations and outpatient services contributed marginally to overall expenditure. In this subgroup, repeated therapeutic changes were associated with a progressive redistribution of drug-related costs across successive treatment lines, reflecting the cumulative impact of treatment instability over time. Similar dynamics have been described in other healthcare systems, where biologic switching represents a relevant determinant of long-term pharmaceutical expenditure in psoriasis management [43]. These findings support the need to optimize therapeutic sequencing strategies, as early selection of more durable treatments may generate meaningful medium- to long-term cost savings for the healthcare system.

These results must be taken with caution in view of some limitations. First, the retrospective observational design precludes causal inference. In addition, administrative databases lack detailed clinical information such as disease severity, including Psoriasis Area and Severity Index (PASI), body surface area (BSA), and patient-reported outcomes such as the Dermatology Life Quality Index (DLQI), limiting the ability to directly assess treatment effectiveness and clinical response. Moreover, although through administrative data it is not possible to make a perfect distinction between psoriasis and psoriatic arthritis—just as, in fact, occurs in real-life clinical practice—no specific clinical exclusion criteria were applied, so that it is possible that some patients could have been affected by both conditions and were receiving biologics also for the other indication. Additionally, in the absence of direct clinical measures, treatment failure was indirectly inferred from treatment switching, which should therefore be interpreted as a proxy rather than a direct measure of inadequate clinical response. Misclassification of comorbidities and treatments might also occur, although validated algorithms were used. Furthermore, the significant baseline differences observed across biologic classes, particularly the higher comorbidity burden and greater prior treatment exposure among patients initiating anti-TNFα agents, suggest potential channeling bias, which may have influenced switching rates and drug survival estimates despite multivariable adjustment. Finally, residual confounding related to physician preference or regional prescribing policies cannot be excluded.

In conclusion, this real-world analysis showed that IL-23 inhibitors were associated with the lowest risk of treatment switching, supporting their superior drug survival compared with other biologic classes. This finding potentially reflects a combination of sustained clinical effectiveness, favorable tolerability, convenient maintenance dosing, and real-world prescribing practices. The association between treatment persistence and reduced HCRU further supports the value of early, effective, and stable first-line biologic strategies within the Italian NHS, while temporal prescribing patterns highlight the influence of market availability and reimbursement policies on real-world treatment choices. Future prospective studies integrating administrative data with clinical outcomes, patient-reported outcome measures, and health–economic evaluations are warranted to confirm these findings, clarify the determinants and biological mechanisms of long-term treatment persistence, including the modulation of the IL-23/Th17 axis and tissue-resident immune response, and identify the patients most likely to benefit from early IL-23 inhibitor initiation. Comparative pragmatic and real-world studies, together with long-term safety and persistence registries, will be essential to define the long-term clinical and economic value of IL-23 inhibitors, including in clinically relevant subgroups such as patients with psoriatic arthritis, metabolic syndrome, or cardiovascular disease.

## Figures and Tables

**Table 1:** Baseline characteristics of adult patients with psoriasis, overall and by type of first-line biologic therapy. Significant *p*-values are highlighted in bold.

	Overall *N* = 10,270	Anti TNFα *N* = 5078	Anti IL-12/23 *N* = 767	Anti IL-17 *N* = 2574	Anti IL-23 *N* = 1851	*p*
Characterization period (years), mean (SD)	8.0 (3.3)	7.9 (3.4)	6.4 (2.7)	7.9 (3.2)	9.0 (3.2)	
Male, *N* (%)	5797 (56.4%)	2580 (50.8%)	454 (59.2%)	1587 (61.7%)	1176 (63.5%)	**<0.001**
Age (mean, SD)	51.9 (14.7)	52.0 (14.3)	51.5 (14.3)	51.8 (14.8)	51.9 (15.9)	0.787
Area						**<0.001**
North, *N* (%)	2708 (26.4%)	1339 (26.4%)	135 (17.6%)	683 (26.5%)	551 (29.8%)	
Center, *N* (%)	2829 (27.5%)	1621 (31.9%)	299 (39.0%)	514 (20.0%)	395 (21.3%)	
South, *N* (%)	4733 (46.1%)	2118 (41.7%)	333 (43.4%)	1377 (53.5%)	905 (48.9%)	
CCI (mean, SD)	0.8 (0.8%)	1.3 (0.6%)	0.3 (0.6%)	0.3 (0.6%)	0.3 (0.6%)	**<0.001**
0, *N* (%)	3868 (37.7%)	0 (0.0%)	554 (72.2%)	1893 (73.5%)	1421 (76.8%)	**<0.001**
1, *N* (%)	4942 (48.1%)	3867 (76.2%)	181 (23.6%)	554 (21.5%)	340 (18.4%)	
≥2, *N* (%)	1460 (14.2%)	1211 (23.8%)	32 (4.2%)	127 (4.9%)	90 (4.9%)	
Comorbidities						
PsA, *N* (%)	1403 (13.7%)	892 (17.6%)	101 (13.2%)	319 (12.4%)	91 (4.9%)	**<0.001**
IBD, *N* (%)	241 (2.3%)	186 (3.7%)	39 (5.1%)	6 (0.2%)	10 (0.5%)	**<0.001**
CV events, *N* (%)	463 (4.5%)	197 (3.9%)	33 (4.3%)	131 (5.1%)	102 (5.5%)	**0.011**
Hypertension, *N* (%)	3992 (38.9%)	1962 (38.6%)	292 (38.1%)	1026 (39.9%)	712 (38.5%)	0.680
Diabetes, *N* (%)	1046 (10.2%)	474 (9.3%)	79 (10.3%)	297 (11.5%)	196 (10.6%)	**0.023**
Cancer, *N* (%)	383 (3.7%)	121 (2.4%)	21 (2.7%)	126 (4.9%)	115 (6.2%)	**<0.001**
Thyroiditis, *N* (%)	62 (0.6%)	32 (0.6%)	5 (0.7%)	14 (0.5%)	11 (0.6%)	0.970
Dyslipidemia, *N* (%)	2018 (19.6%)	962 (18.9%)	171 (22.3%)	501 (19.5%)	384 (20.7%)	0.091
Depression, *N* (%)	1102 (10.7%)	616 (12.1%)	80 (10.4%)	242 (9.4%)	164 (8.9%)	**<0.001**
Previous and other treatments						
Conventional systemic drugs, *N* (%)	4958 (48.3%)	2830 (55.7%)	342 (44.6%)	1147 (44.6%)	639 (34.5%)	**<0.001**
Antipsoriatics for topical use, *N* (%)	6300 (61.3%)	2816 (55.5%)	453 (59.1%)	1730 (67.2%)	1301 (70.3%)	**<0.001**
Number of co-treatments (mean, SD)	7.5 (5.6)	8.0 (5.7)	7.4 (5.7)	7.3 (5.7)	6.5 (5.2)	**<0.001**
0–4, *N* (%)	3695 (36.0%)	1623 (32.0%)	287 (37.4%)	969 (37.6%)	816 (44.1%)	**<0.001**
5–9, *N* (%)	3552 (34.6%)	1801 (35.5%)	253 (33.0%)	873 (33.9%)	625 (33.8%)	
≥10, *N* (%)	3023 (29.4%)	1654 (32.6%)	227 (29.6%)	732 (28.4%)	410 (22.2%)	

SD: standard deviation; CCI: Charlson Comorbidity Index; PsA: psoriatic arthritis; IBD: inflammatory bowel disease; CV: cardiovascular.

**Table 2:** Distribution of first-line treatments (grouped by class) by index year.

	2015	2016	2017	2018	2019	2020	2021	2022	2023	2024
TNFα inhibitors	80.1%	68.8%	49.8%	36.4%	41.1%	42.6%	45.1%	48.8%	50.5%	68.2%
IL-12/23 inhibitor	19.9%	24.4%	21.9%	19.7%	12.3%	6.2%	2.6%	1.5%	1.3%	1.6%
IL-17 inhibitors	0.0%	6.8%	28.3%	43.8%	38.5%	30.9%	26.2%	21.7%	21.1%	14.6%
IL-23 inhibitors	0.0%	0.0%	0.0%	0.0%	8.0%	20.2%	26.3%	28.0%	27.2%	15.5%

**Table 3:** Distribution of second-line biologic therapies according to first-line class among patients who switched treatment (*N* = 2743).

First-Line Therapy (Class), *N* = 10,270	Second-Line Therapy (Class)—Switch, *N* = 2743			
	TNFα Inhibitor	IL12/23 Inhibitor	IL17 Inhibitor	IL23 Inhibitor
TNFα inhibitor (*N* = 5078)	582 (11.5%)	183 (12.5%)	852 (58.3%)	427 (29.2%)
IL12/23 inhibitor (*N* = 767)	68 (22.4%)	-	80 (26.4%)	155 (51.2%)
IL17 inhibitor (*N* = 2574)	173 (32.9%)	21 (4%)	164 (6.4%)	332 (63.1%)
IL23 inhibitor (*N* = 1851)	20–30%	NI	53 (70.7%)	18 (1%)

NI: not issuable; Percentage ranges reported refer only to the specific sub-analysis and are omitted for privacy reasons (i.e., to limit the risk of indirectly referring to single patients).

**Table 4:** Cox regression model for predictors of switching (anti-TNF agents as the reference category). Significant *p*-values are in bold.

Outcome: Switch	HR	95% CI		*p*-Value
Index drug (Ref.: Anti-TNF)	1.000			
Anti-IL17	0.641	0.558	0.736	**<0.001**
Anti-IL12/23	0.915	0.783	1.069	0.263
Anti-IL23	0.186	0.144	0.240	**<0.001**
Age	0.995	0.992	0.999	**0.011**
Male (Ref.: female)	0.893	0.819	0.973	**0.010**
CCI	1.045	0.951	1.149	0.362
PsA (Ref.: absence)	1.057	0.945	1.183	0.330
IBD (Ref.: absence)	0.622	0.453	0.854	**0.003**
CV events (Ref.: absence)	0.903	0.718	1.134	0.380
Hypertension (Ref.: absence)	1.081	0.972	1.203	0.152
Diabetes (Ref.: absence)	1.021	0.859	1.213	0.815
Dyslipidemia (Ref.: absence)	1.021	0.904	1.153	0.736
Depression (Ref.: absence)	1.183	1.037	1.350	**0.012**
Number of conventional systemic drugs (Ref.: 0)	1.000			
1–2	1.201	1.102	1.309	**<0.001**
≥3	2.305	1.357	3.915	**0.002**
Antipsoriatics for topical use (Ref.: absence)	1.236	1.132	1.349	**<0.001**
Number of co-treatments (Ref.: 0–4)	1.000			
5–9	0.958	0.860	1.067	0.437
≥10	0.880	0.766	1.011	0.071

CCI: Charlson Comorbidity Index; PsA: psoriatic arthritis; IBD: inflammatory bowel disease; CV: cardiovascular; HR: hazard ratio; CI: confidence interval.

**Table 5:** Cox regression model for predictors of switching (anti-IL12/23 agents as the reference category). Significant *p*-values are in bold.

Outcome: Switch	HR	95% CI		*p*-Value
Index drug (Ref.: Anti-IL12/23)	1.000			
Anti-IL17	0.698	0.605	0.806	**<0.001**
Anti-IL23	0.202	0.156	0.261	**<0.001**
Anti-TNF	1.088	0.931	1.271	0.288
Age	0.995	0.992	0.999	**0.011**
Male (Ref.: female)	0.893	0.819	0.973	**0.010**
CCI	1.043	0.949	1.147	0.380
PsA (Ref.: absence)	1.058	0.946	1.184	0.324
IBD (Ref.: absence)	0.622	0.453	0.854	**0.003**
CV events (Ref.: absence)	0.901	0.717	1.133	0.373
Hypertension (Ref.: absence)	1.081	0.971	1.203	0.154
Diabetes (Ref.: absence)	1.022	0.860	1.215	0.802
Dyslipidemia (Ref.: absence)	1.020	0.903	1.152	0.747
Depression (Ref.: absence)	1.180	1.034	1.346	**0.014**
Conventional systemic drugs (Ref.: absence)	1.207	1.107	1.315	**<0.001**
Antipsoriatics for topical use (Ref.: absence)	1.236	1.133	1.350	**<0.001**
Number of co-treatments (Ref.: 0–4)	1.000			
5–9	0.960	0.862	1.070	0.461
≥10	0.883	0.769	1.015	0.080

CCI: Charlson Comorbidity Index; PsA: psoriatic arthritis; IBD: inflammatory bowel disease; CV: cardiovascular; HR: hazard ratio; CI: confidence interval.

**Table 6:** Healthcare resource utilization (HCRU) and associated direct medical costs during the first 12 months after biologic initiation, stratified by switching status (switchers vs. non-switchers) and biologic class. Significant *p*-values are highlighted in bold.

	TNFα Inhibitors			IL12/23 Inhibitor (Ustekinumab)			IL17 Inhibitors			IL23 Inhibitors		
	Non-Switchers (*N* = 2749)	Switchers (*N* = 1387)	*p* Value	Non-Switchers (*N* = 419)	Switchers (*N* = 299)	*p* Value	Non-Switchers (*N* = 6152)	Switchers (*N* = 2258)	*p* Value	Non-Switchers (*N* = 1318)	Switchers (*N* = 71)	*p* Value
**HCRU (mean number of healthcare resources utilized per patient, SD)**												
Any drug prescriptions	18.8 (11.3)	18.4 (10.3)	0.357	13.1 (9.1)	15.6 (10.5)	**0.001**	16.6 (10.9)	17.9 (10.4)	<0.001	13.0 (9.9)	15.9 (10.5)	0.018
TNFα inhibitors	7.5 (3.5)	6.4 (3.5)	**<0.001**	0.0 (0.0)	0.2 (0.9)	**<0.001**	3.4 (4.4)	4.1 (4.1)	<0.001	0.0 (0.0)	0.4 (1.6)	**<0.001**
IL12/23 inhibitors	0.0 (0.0)	0.1 (0.6)	**<0.001**	4.4 (1.4)	4.3 (1.4)	0.374	0.3 (1.2)	0.7 (1.6)	<0.001	0.0 (0.0)	0.0 (0.1)	**<0.001**
IL17 inhibitors	0.0 (0.0)	0.9 (1.9)	**<0.001**	0.0 (0.0)	0.2 (1.0)	**<0.001**	1.9 (3.5)	2.1 (3.3)	0.026	0.0 (0.0)	1.2 (2.1)	**<0.001**
IL 23 inhibitors	0.0 (0.0)	0.3 (1.0)	**<0.001**	0.0 (0.0)	0.0 (0.3)	**0.025**	1.0 (2.1)	0.4 (1.1)	<0.001	4.7 (1.6)	3.6 (2.0)	**<0.001**
Hospitalizations	0.1 (0.4)	0.1 (0.5)	0.350	0.1 (0.4)	0.2 (0.4)	0.648	0.1 (0.4)	0.1 (0.5)	0.008	0.1 (0.4)	0.1 (0.4)	0.304
OSS	6.7 (6.1)	6.6 (6.2)	0.632	4.9 (5.1)	5.7 (6.1)	0.034	5.9 (5.8)	6.5 (6.3)	<0.001	5.1 (5.2)	6.6 (6.9)	**0.019**
**Mean direct medical costs per patient (€)**												
Any drug dispensations	4729.0	7788.1	**<0.001**	12,740.1	13,164.3	0.128	8489.4	9599.8	**<0.001**	10,865.0	11,284.6	0.274
TNFα inhibitors	4009.6	4471.8	**0.001**	0.0	230.8	**<0.001**	1791.7	2837.9	**<0.001**	0.0	89.6	**<0.001**
IL12/23 inhibitors	0.0	364.7	**<0.001**	12,243.4	11,887.6	0.214	833.9	1828.5	**<0.001**	0.0	38.8	**<0.001**
IL17 inhibitors	0.0	1552.0	**<0.001**	0.0	452.2	**<0.001**	3047.4	3407.5	**0.006**	0.0	2271.3	**<0.001**
IL 23 inhibitors	0.0	781.4	**<0.001**	0.0	70.0	0.021	2234.1	913.4	**<0.001**	10,427.9	8013.4	**<0.001**
Hospitalizations	388.3	406.9	0.746	219.8	227.9	0.912	319.4	365.6	0.211	273.1	341.1	0.677
OSS	329.0	324.5	0.823	286.3	253.5	0.542	300.7	318.8	0.233	267.9	427.3	**0.016**
Total	5446.3	8519.4	**<0.001**	13,246.2	13,645.7	0.171	9109.5	10,284.3	**<0.001**	11,406.0	12,052.9	0.132

HCRU: healthcare resource utilization; SD: standard deviation; OSS: outpatient specialist services.

**Table 7:** Pattern of treatment sequence in multifailure patients (*N* = 750).

**First-line therapy (class), *N* = 750**	**Second-line therapy (class)**			
	**TNFα inhibitor**	**IL12/23 inhibitor**	**IL17 inhibitor**	**IL123 inhibitor**
TNFα inhibitor, *N* = 481	*N* = 182 (37.8%)	*N* = 40 (8.3%)	*N* = 193 (40.1%)	*N* = 66 (13.7%)
IL12/23 inhibitor, *N* = 81	*N* = 31 (38.3%)	/	*N* = 36 (44.4%)	*N* = 14 (17.3%)
IL17 inhibitor, *N* = 177	*N* = 62 (35.0%)	*N* = 11 (6.2%)	*N* = 64 (36.2%)	*N* = 40 (22.6%)
IL23 inhibitor, *N* = 11	*N* = 6 (54.5%)	*N* = 5 (45.5%)	*N* = 0 (0.0%)	*N* = 0 (0.0%)
**Second-line therapy (class)**	**Third-line therapy (class)**			
	**TNFα inhibitor**	**IL12/23 inhibitor**	**IL17 inhibitor**	**IL123 inhibitor**
TNFα inhibitor, *N* = 281	*N* = 37 (13.2%)	*N* = 18 (6.4%)	*N* = 163 (58.0%)	*N* = 63 (22.4%)
IL12/23 inhibitor, *N* = 77	*N* = 15 (19.5%)	*N* = 0 (0.0%)	*N* = 37 (48.0%)	*N* = 25 (32.5%)
IL17 inhibitor, *N* = 298	*N* = 63 (21.1%)	*N* = 19 (6.4%)	*N* = 58 (19.5%)	*N* = 158 (53.0%)
IL23 inhibitor, *N* = 94	10–15%	*N* = NI	*N* = 56 (59.6%)	*N* = 25 (26.6%)

NI: not issuable; Percentage ranges reported refer only to the specific sub-analysis and are omitted for privacy reasons (i.e., to limit the risk of indirectly referring to single patients).

**Table 8:** HCRU and associated direct costs after start of the first biologic treatment during the entire available follow-up, overall and by class, among multifailure patients. Significant *p*-values are highlighted in bold.

	Overall	TNFα Inhibitors	IL12/23 Inhibitors	IL17 Inhibitors	IL23 Inhibitors	*p*-Value
*N* Patients *	741	473	81	176	11	
**HCRU (mean number of healthcare resources utilized per patient, SD)**						
Drugs (any)	17.9 (9.9)	18.2 (9.9)	16.8 (8.5)	17.9 (10.5)	14.7 (8.5)	0.481
Hospitalizations	0.2 (0.3)	0.2 (0.3)	0.1 (0.2)	0.2 (0.3)	0.2 (0.3)	0.637
OSS	7.1 (5.9)	7.2 (5.8)	5.7 (4.1)	7.5 (6.7)	6.4 (3.6)	0.103
**Mean direct medical costs per patient (€)**						
Drugs (any)	8451.0	8021.9	9147.0	9236.8	9203.8	**<0.001**
Hospitalizations	523.2	521.2	340.7	625.9	307.8	0.530
OSS	386.9	400.8	289.9	393.1	403.4	0.235
Total	9361.0	8943.8	9777.6	10,255.9	9914.9	**<0.001**

* Patients with at least 12 months of follow-up available, excluding deaths and outliers. HCRU: healthcare resource utilization; SD: standard deviation; OSS: outpatients specialist service.

## Data Availability

The data supporting the findings of this article are available at aggregated level from the authors upon reasonable request and with permission of the participating healthcare entities. Requests to access should be directed to the corresponding author.
